# Increased Cellular Uptake of Polyunsaturated Fatty Acids and Phytosterols from Natural Micellar Oil

**DOI:** 10.3390/nu12010150

**Published:** 2020-01-05

**Authors:** Clemens Röhrl, Flora Stübl, Martin Maier, Bettina Schwarzinger, Clemens Schwarzinger, Johannes Pitsch, Peter Lanzerstorfer, Marcus Iken, Julian Weghuber

**Affiliations:** 1 Center of Excellence Food Technology and Nutrition, University of Applied Sciences Upper Austria, 4600 Wels, Austria; flora.stuebl@fh-wels.at (F.S.); bettina.schwarzinger@fh-wels.at (B.S.); johannes.pitsch@fh-wels.at (J.P.); peter.lanzerstorfer@fh-wels.at (P.L.); 2 Austrian Competence Center for Feed and Food Quality, Safety and Innovation, 4600 Wels, Austria; 3 Institute for Chemical Technology of Organic Materials, Johannes Kepler University, 4040 Linz, Austria; clemens.schwarzinger@jku.at; 4 PM International AG, 15 Wäistrooss Schengen, Luxembourg 5445, Luxembourg

**Keywords:** Micellization, algae oil, DHA, PUFAs, phytosterols, fatty acid, cellular uptake

## Abstract

The transport of hydrophobic compounds to recipient cells is a critical step in nutrient supplementation. Here, we tested the effect of phospholipid-based emulsification on the uptake of hydrophobic compounds into various tissue culture cell lines. In particular, the uptake of ω-3 fatty acids from micellar or nonmicellar algae oil into cell models for enterocytes, epithelial cells, and adipocytes was tested. Micellization of algae oil did not result in adverse effects on cell viability in the target cells. In general, both micellar and nonmicellar oil increased intracellular docosahexaenoic acid (DHA) levels. However, micellar oil was more effective in terms of augmenting the intracellular levels of total polyunsaturated fatty acids (PUFAs) than nonmicellar oil. These effects were rather conserved throughout the cells tested, indicating that fatty acids from micellar oils are enriched by mechanisms independent of lipases or lipid transporters. Importantly, the positive effect of emulsification was not restricted to the uptake of fatty acids. Instead, the uptake of phytosterols from phytogenic oils into target cells also increased after micellization. Taken together, phospholipid-based emulsification is a straightforward, effective, and safe approach to delivering hydrophobic nutrients, such as fatty acids or phytosterols, to a variety of cell types in vitro. It is proposed that this method of emulsification is suitable for the effective supplementation of numerous hydrophobic nutrients.

## 1. Introduction

The lipophilic nature of fatty acids and unsaponifiable lipids poses a major technological challenge for the food industry. Lipophilic compounds relevant to food supplementation include fat-soluble vitamins; secondary plant metabolites, such as polyphenols and phytosterols; and polyunsaturated fatty acids (PUFAs), including ω-3 fatty acids [1].

It is generally accepted that ω-3 fatty acids exert numerous beneficial effects on human health. Despite inconclusive evidence for the reduction of mortality, ω-3 fatty acids prevent hyperlipidemia [2], exert anti-inflammatory properties [3], and are indispensable for brain development [4]. While fish oils are rich in ω-3 fatty acids, there is increasing demand for alternative sources from environmentally sustainable and non-animal origins. Algae oils are an alternative and vegan resource of ω-3 fatty acids—especially eicosapentaenoic acid (EPA) and docosahexaenoic acid DHA—and are superior over fish oils in terms of sustainability as well as sensory properties [5]. In addition, algae oils were shown to mimic the beneficial effects of fish oils. Specifically, oil of the marine alga *Schizochytrium* sp. has been used to prevent abdominal fat accumulation in mice [6], mediate anti-inflammatory effects in patients with rheumatoid arthritis [7], and improve learning in a canine model [8].

Phytosterols are plant-derived cholesterol analogs and are known to lower plasma low-density lipoprotein (LDL)-cholesterol levels by competitive inhibition of cholesterol absorption [9]. In addition, the anti-inflammatory properties of phytosterols have been proposed [10]. Indeed, phytosterol-fortified beverages decrease the activity of pro-inflammatory signaling pathways in human subjects without hyperlipidemia [11,12]. Furthermore, phytosterols were found to be beneficial in a murine model of experimental colitis [13], altogether demonstrating that phytosterols have the potential to modulate inflammatory diseases beyond LDL-cholesterol reduction. To deliver these and other lipophilic compounds to desired target cells, various methods, including emulsification [14], microencapsulation [15], and gelled emulsion [16], have been applied. For instance, soya lecithin improved bioavailability of α-linolenic acid [17] in rats. Similarly, crude lecithin increased the bioavailability of DHA from fish and vegetable oil in rats [18].

In this study, we investigated the efficacy of oil emulsification by phospholipid-based micellization for the delivery of ω-3 fatty acids from algae oil derived from *Schizochytrium* sp. and phytosterols from phytogenic oil to recipient cells using a variety of human cell models. Specifically, cell models for enterocytes, epithelial cells, and adipocytes were chosen for this study with respect to their different capabilities of processing and transporting lipophilic compounds. Our results provide evidence for the increased uptake of fatty acids and phytosterols from micellar phytogenic oil compared to that of nonmicellar phytogenic oil, which was largely independent of the respective cellular model system.

## 2. Materials and Methods

### 2.1. Cell Culture

Cells were maintained under standard conditions and routinely checked for mycoplasma infections. Cell culture reagents were obtained from Biochrom GmbH (Berlin, Germany). Caco-2 cells (HTB-37; ATCC, City of Manassas, VA, USA) were maintained in Minimum Essential Media with Earle’s salts supplemented with penicillin/streptomycin and 10% FBS. For differentiation, cells were grown until confluency and then incubated with Entero-STIM intestinal epithelium differentiation medium supplemented with 0.1% MITO + serum extender (all from Corning, Wiesbaden, Germany) and penicillin/streptomycin. Cells were used for experiments after five days of differentiation.

3T3-L1 cells (CL-173, ATCC) were maintained in Dulbecco’s modified Eagle’s medium (DMEM) supplemented with penicillin/streptomycin and 10% FBS as previously reported [19]. For differentiation, cells were grown until confluency and cultivated for another five days, and the media was exchanged twice. Afterward, cells were incubated with differentiation media (DMEM containing 10% FBS and penicillin/streptomycin supplemented with 0.25 µmol/L dexamethasone, 10 µg/mL insulin, and 500 µmol/L 3-isobutyl-1-methylxanthine (IBMX); all from Sigma-Aldrich, Schnelldorf, Germany) for three days. Cells were grown in post-differentiation media (DMEM containing 10% FBS and penicillin/streptomycin supplemented with 10 µg/mL insulin) for another seven days, and the media was exchanged two times prior to use for experiments.

MDCK.2 cells (CRL-2936, ATCC) were maintained in Minimum Essential Media with Earle’s salts supplemented with penicillin/streptomycin and 10% FBS.

OP9 cells (CRL-2749; ATCC) were maintained in Alpha Minimum Essential Medium without ribonucleotides and deoxyribonucleotides supplemented with sodium bicarbonate (2.2 g/L), penicillin/streptomycin, and 20% FBS. For differentiation, cells were grown until confluency and cultivated in DMEM supplemented with 10% FBS and penicillin/streptomycin for another two days. Afterwards, cells were incubated in differentiation media (see above) for three days and in post-differentiation media (see above) for another four days.

### 2.2. Uptake of Fatty Acids from Algae Oils

Cells were seeded into 15-cm dishes and differentiated as described above when indicated. Micellar oil containing 10% algae oil derived from *Schizochytrium* sp. was emulsified in water with glycerol and soy-phospholipids and was obtained from Bio-Gen (Montabaur, Germany) and diluted in DMEM + 10% FBS to a final concentration of 1% or 0.5% for experiments using Caco-2 cells or 3T3-L1 and OP9 cells, respectively. Nonmicellar, pure algae oil (Bio-Gen) was diluted 1/10 in 70% ethanol and was further diluted in DMEM + 10% FBS to a final concentration of 1% or 0.5% for experiments using Caco-2 or 3T3-L1 cells, respectively. After 6 h of incubation, cells were washed four times with PBS, detached using trypsin, and resuspended in DMEM + 10% FBS. Cells were centrifuged (4 °C, 200× *g*, 5 min), and cell pellets were washed with ddH_2_O followed by another step of centrifugation. Cell pellets were dried in a vacuum centrifuge and further processed for fatty-acid methyl ester (FAME) analysis.

### 2.3. FAME Analysis

For transesterification, samples were mixed with 200 µL methanolic sodium hydroxide (0.5 mol/L; Sigma-Aldrich, St. Louis, MO, USA) and incubated at 60 °C for 45 min while shaking. Next, 300 µL methanolic boron trifluoride solution (1.3 mol/L; Sigma-Aldrich, St. Louis, MO, USA) was added, and incubation was continued for another 30 min. The reaction was stopped by the addition of 200 µL saturated sodium chloride solution. FAMEs were extracted with n-hexane and subjected to GC-MS analysis. Capillary GC-MS analysis was performed using a Trace 1300 GC with an ISQ QD mass selective detector and a PTV injector (Thermo Fisher Scientific, Waltham, MA, USA). One microliter of sample was separated using a DB-23 column (60 m × 0.25 mm, film thickness: 0.25 µm; Agilent, Santa Clara, CA, USA) and a constant helium carrier gas flow of 1.5 mL/min. The injector temperature was set to 240 °C. The oven temperature program was as follows: 130 °C for 5 min; heating to 170 °C at 6.5 °C/min; heating to 215 °C at 1.50 °C/min; 215 °C for 12 min; heating to 240 °C at 5.0 °C/min; and 240 °C for 10 min. The mass spectra were recorded over 40–500 *m*/*z*. Quantification was carried out using external calibration with specific FAME standards in the range of 5–400 µg/mL. The amount of individual fatty acids was determined relative to the total fatty acid content.

### 2.4. Cell Viability Assay

Cells were seeded into 96-well plates, and differentiation was initiated as described above. Cells were treated with micellar and nonmicellar oils in the same concentrations as used for the uptake assays. After 24 h, cell viability was assessed by measuring metabolically active cells using a resazurin-based in vitro toxicology assay kit (Sigma-Aldrich, Schnelldorf, Germany).

### 2.5. Uptake of Phytosterol from Plant Oils

Caco-2 cells were seeded into 15-cm dishes and differentiated as described above. MDCK.2 cells were seeded into 15-cm dishes and cultivated until confluency was achieved. Micellar phytogenic oil containing 3% oil emulsified in water with glycerol and soy-phospholipids (Bio-Gen) as well as nonmicellar phytogenic oil containing 3% oil and glycerol only (Bio-Gen) were diluted 1:4 in DMEM + 10% FBS, resulting in a final concentration of 0.75% oil. During incubation, cells were gently agitated every hour. After 6 h of incubation, cells were washed four times with PBS, detached using trypsin and resuspended in DMEM + 10% FBS. Cells were centrifuged (4 °C, 200× *g*, 5 min), and the cell pellets were washed with ddH_2_O followed by another step of centrifugation. Cell pellets were dried in a vacuum centrifuge and further processed for phytosterol analytics.

### 2.6. Phytosterol Analytics

Dried cell pellets were weighed and incubated with 600 µL ethanolic sodium hydroxide (2 mol/L) at 80 °C for 1 h to hydrolyze phytosterol-esters. After the addition of 300 µL ddH_2_O, phytosterols were extracted with hexane, evaporated, dissolved in chloroform, and subjected to HPLC-MS analysis. Analyses were carried out using a Surveyor HPLC (Thermo Fisher Scientific, Waltham, MA, USA) attached to an Orbitrap Velos (Thermo Fisher Scientific, Waltham, MA, USA) mass spectrometer, which was operated in ion trap mode as previously reported [20]. Ionization was achieved via an atmospheric pressure chemical ionization (APCI) ion source in positive mode, and the *m*/*z* ratios of campesterol (383.3 ± 0.5), stigmasterol (395.4 ± 0.5), and β-sitosterol (397.4 ± 0.5) were recorded in SIM mode. Compounds were separated on an Accucore C18 column (150 mm × 3 mm, 2.6 µL; Thermo Fisher Scientific, Waltham, MA, USA) at 40 °C using a ternary gradient of methanol/acetonitrile with 0.1% formic acid/2-propanol (75/20/5 for 23 min; 20/70/10 for 4 min; and 75/20/5 for 3 min). Quantification was carried out using external calibration with campesterol, stigmasterol, and β-sitosterol (Sigma Aldrich, St. Louis, MO, USA), and the phytosterol concentrations were normalized to cell dry mass.

### 2.7. Statistics

Data are expressed as the mean ± SD. Statistical analysis was performed using GraphPad Prism (GraphPad Software, San Diego, CA, USA; version 8.0.2). Two-sided *t*-tests were applied to compare two experimental groups. ANOVA followed by Tukey’s multiple testing corrections was used to compare more than two experimental groups. Significant *p*-values are indicated as * (≤0.05), ** (≤0.01), or *** (≤0.001).

## 3. Results

### 3.1. Analysis of Fatty Acid Composition of Algae Oil

To study the effect of micellization on the delivery of fatty acids to recipient cells, algae oils were either used in their native, nonmicellar form (“oil”) or after micellization (“micellar oil”). Both preparations were comparable in terms of their total fatty acid content (Table 1), with oleic acid, palmitic acid, docosapentaenoic acid (DPA), and DHA quantitatively being the most important fatty acids. Algae oils consisted of ~64% DHA.

### 3.2. Uptake of Algae Oil into Cell Models of Enterocytes

Different cell models were incubated with the oil preparations, and the enrichment of fatty acids in the target cells was analyzed. In initial experiments, palmitic acid, palmitoleic acid, stearic acid, cis-9-oleic acid, linoleic acid, α-linolenic acid, arachidonic acid, EPA, DPA, and DHA were identified as the most abundant relevant fatty acids in the cell lines used; therefore, the content of these fatty acid species was analyzed in detail.

**Figure 1:**
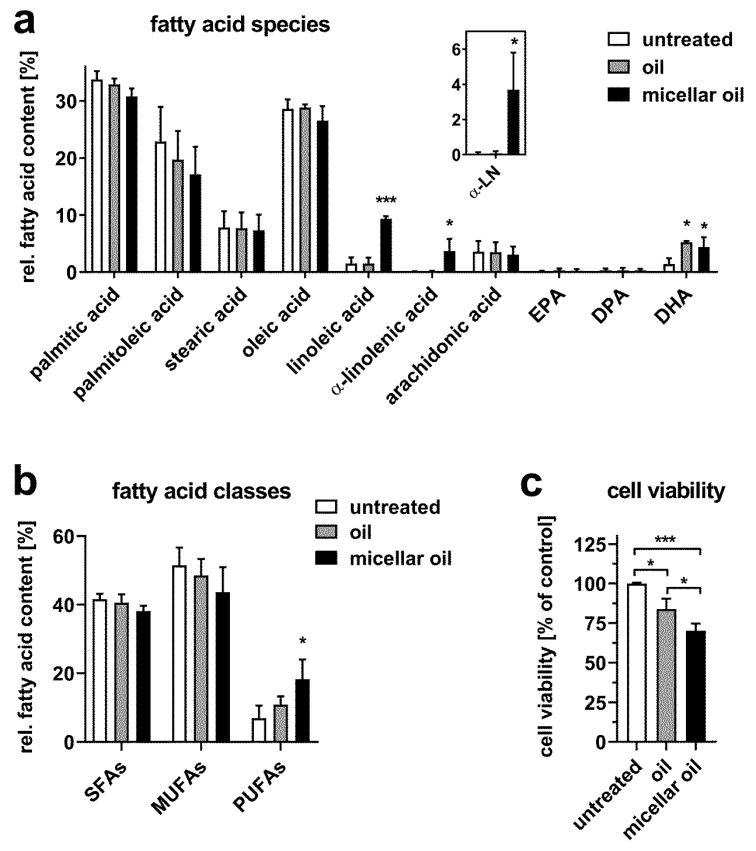
Uptake of fatty acids from algae oil into differentiated Caco-2 cells. Cells were incubated with native or micellar oils at a final concentration of 1% for 6 h. Total fatty acid content was measured by GC-MS. The relative fatty acid composition (**a**) and distribution of fatty acid classes (**b**) are shown. The inset indicates an enlarged view on α-linolenic acid (α-LN) levels. Cell viability was assessed after incubation with 1% oil or micellar oil for 24 h (**c**). Data are derived from three independent experiments. Bars represent mean ± SD. Asterisks indicate statistically significant differences from untreated controls. *** *p* < 0.001 and * *p* < 0.05.

To mimic fatty acid uptake in the intestine, human Caco-2 cells were differentiated to resemble enterocytes of the small intestine [21,22]. Figure 1a shows that incubation of cells with nonmicellar bulk oil increased the content of cellular DHA. The micellar oil likewise increased the DHA content as well as the linoleic and α-linolenic acid contents. Only the micellar oil augmented the total content of cellular PUFAs (Figure 1b). Both oil preparations slightly decreased cell viability. This decrease was more pronounced after incubation with micellar oil compared to that after incubation with nonmicellar oil (Figure 1c).

Differentiated Caco-2 cells are known to resecrete fatty acids after the formation of chylomicron-like particles compared to native enterocytes. We therefore tested whether the observed increase in PUFAs by micellar oil was a consequence of altered fatty acid uptake or resecretion. Therefore, nondifferentiated Caco-2 cells cultivated under standard conditions, which do not secrete chylomicron-like particles [23], were used as a model system.

**Figure 2:**
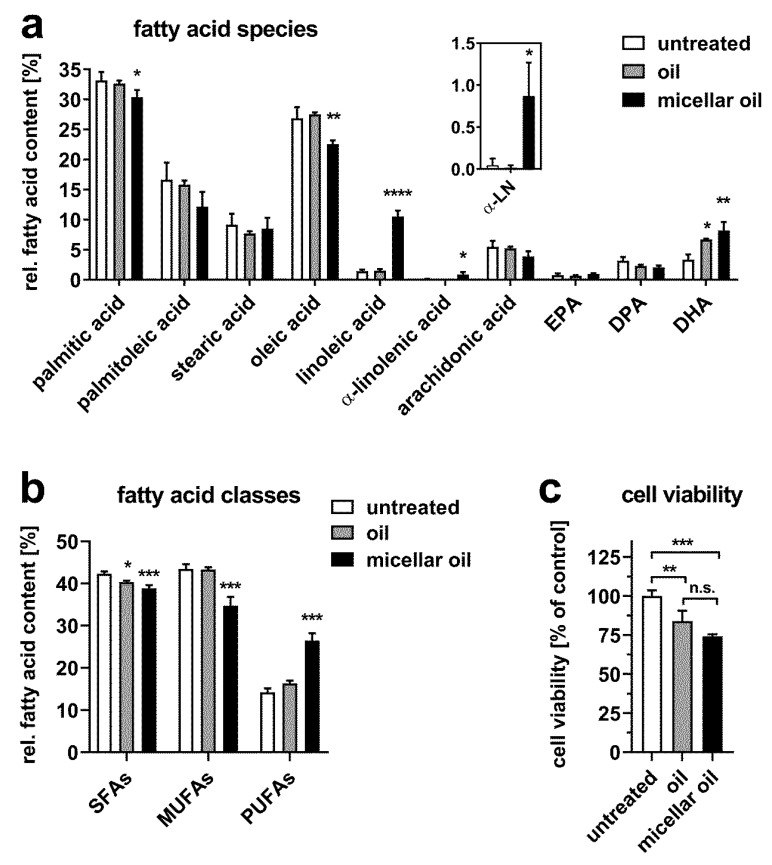
Uptake of fatty acids from algae oil into nondifferentiated Caco-2 cells. Cells incubated with native or micellar oils at a final concentration of 1% for 6 h. The total fatty acid content was measured by GC-MS. The relative fatty acid composition (**a**) and distribution of fatty acid classes (**b**) are shown. The inset indicates an enlarged view on α-linolenic acid (α-LN) levels. Cell viability was assessed after incubation with 1% oil or micellar oil for 24 h (**c**). Data are derived from three independent experiments. Bars represent mean ± SD. Asterisks indicate statistically significant differences from untreated controls. **** *p* < 0.0001, *** *p* < 0.001, ** *p* < 0.01 and * *p* < 0.05.

Similar to differentiated Caco-2 cells, both the micellar and nonmicellar oils elevated relative DHA levels, but only incubation with micellar oil increased linoleic and α-linolenic acid content (Figure 2a). In contrast, micellar oil reduced the relative concentrations of palmitic and oleic acid. Again, only the micellar oil increased the total PUFA content (Figure 2b). Cell viability was slightly reduced by both oil preparations and did not differ between micellar and nonmicellar oil (Figure 2c). These data indicate that micellar oils increase cellular PUFA content because of increased uptake rather than by modifying resecretion.

### 3.3. Uptake of Algae Oil into Adipocyte Cell Models

We then aimed to analyze whether the uptake of micellar oil relies on extracellular lipase activity. Caco-2 cells possess limited extracellular lipase activity [24]. In contrast, adipocytes express and secrete lipoprotein lipase (LPL), which is capable of hydrolyzing free fatty acids from triglyceride-rich particles [25]. Thus, 3T3-L1 cells were differentiated into adipocytes, which represents a widely used adipocyte model that is known to express both LPL and its activator apolipoprotein C-II [26].

**Figure 3:**
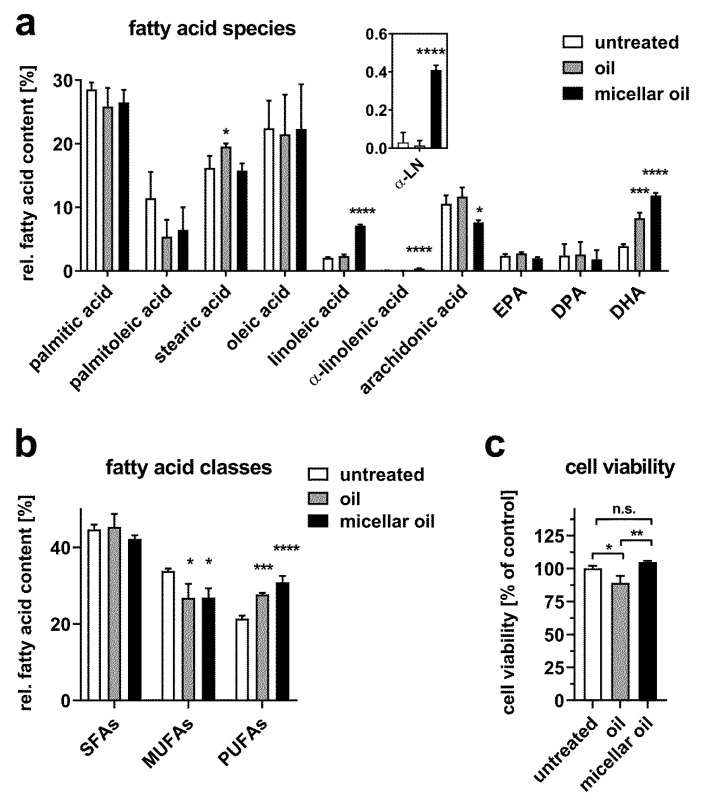
Uptake of fatty acids from algae oil into 3T3-L1 adipocytes. Cells were incubated with native or micellar oils at a final concentration of 0.5% for 6 h. Total fatty acid content was measured by GC-MS. Relative fatty acid composition (**a**) and distribution of fatty acid classes (**b**) are shown. The inset indicates an enlarged view on α-linolenic acid (α-LN) levels. Cell viability was assessed after incubation with 0.5% oil or micellar oil for 24 h (**c**). Data are derived from three independent experiments. Bars represent mean ± SD. Asterisks indicate statistically significant differences from untreated controls. **** *p* < 0.0001, *** *p* < 0.001, ** *p* < 0.01 and * *p* < 0.05.

Experiments with 3T3-L1 adipocytes yielded results comparable to those obtained with the enterocyte model: while the nonmicellar oil increased cellular DHA content only, the micellar oil augmented the levels of DHA, α-linolenic acid, and linoleic acid (Figure 3a). In parallel, the micellar oil decreased relative arachidonic acid levels. In contrast to the enterocyte model, however, both oil preparations increased cellular PUFA content significantly, whereas the MUFA content declined (Figure 3b). A slight reduction in cell viability was observed only after incubation with nonmicellar oil (Figure 3c).

**Figure 4:**
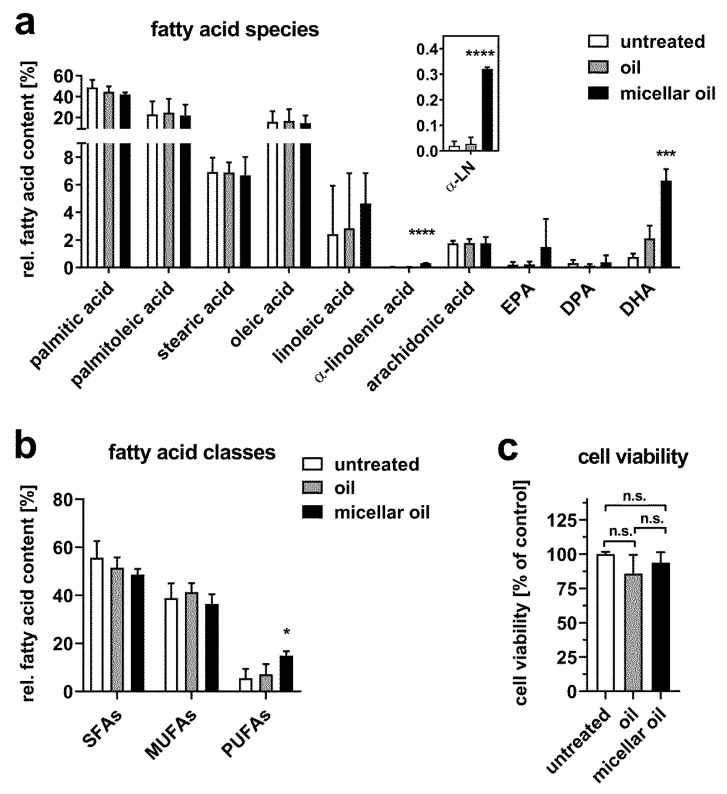
Uptake of algae oil into OP9 adipocytes. Cells were incubated with native or micellar oils at a final concentration of 0.5% for 6 h. Total fatty acid content was measured by GC-MS. Relative fatty acid composition (**a**) and distribution of fatty acid classes (**b**) are shown. The inset indicates an enlarged view on α-linolenic acid (α-LN) levels. Cell viability was assessed after incubation with 0.5% oil or micellar oil for 24 h (**c**). Data are derived from three independent experiments. Bars represent mean ± SD. Asterisks indicate statistically significant differences from untreated controls. **** *p* < 0.0001, *** *p* < 0.001 and * *p* < 0.05.

In addition to 3T3-L1 cells, we investigated the uptake of micellar oil in another adipocyte cell model: OP9 mouse stromal cells can be differentiated into adipocytes and are frequently used as a model of adipogenesis [27]. Similar to 3T3-L1, OP9 cells express high levels of LPL [28]. As shown in Figure 4, only the micellar oil increased cellular DHA, α-linolenic acid, and PUFA content significantly, whereas the MUFA concentration remained unchanged. In contrast, the nonmicellar oil did not change any of the aforementioned parameters significantly. No effect on cell viability was observed for either micellar or non-micellar oil (Figure 4c).

### 3.4. Uptake of Phytosterols from Phytogenic Oil

Finally, we tested whether the effect of micellization is restricted to fatty acid uptake or if it is more general in nature and likewise affects the cellular delivery of other lipophilic compounds. Therefore, the uptake of phytosterols from phytogenic oil into recipient cells was analyzed using either native or micellar phytogenic oil.

**Figure 5:**
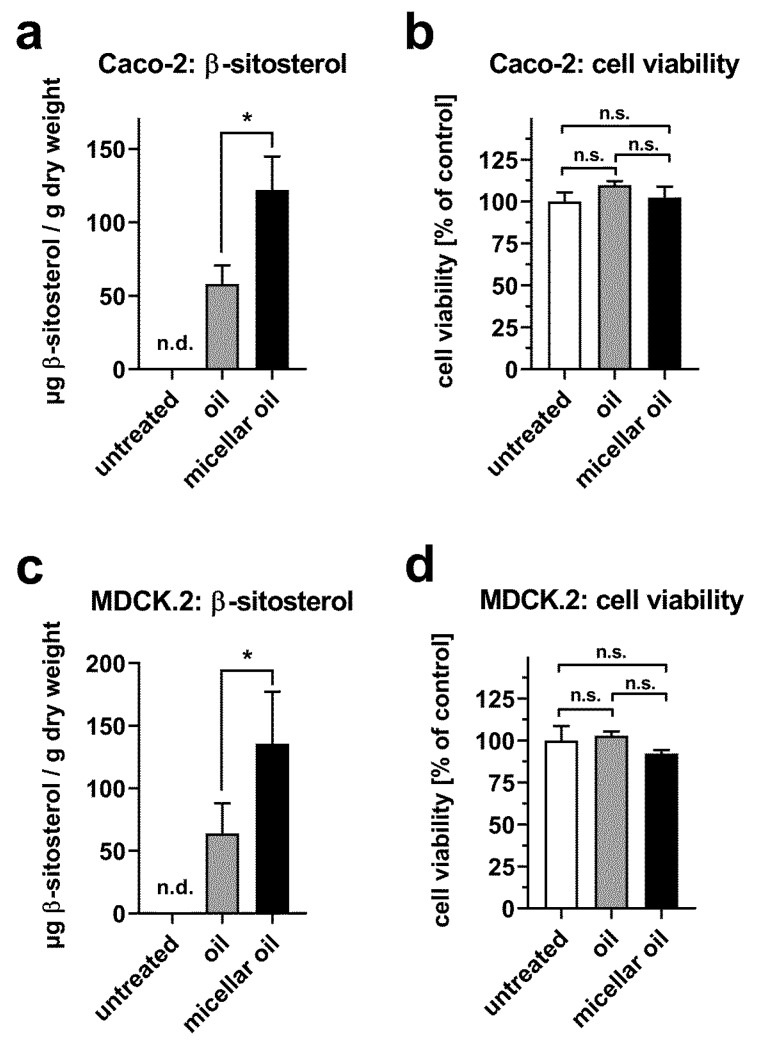
Uptake of phytosterols from phytogenic oil. Differentiated Caco-2 cells (**a**) or MDCK.2 cells (**c**) were incubated with native or micellar oils at a final concentration of 0.75% for 6 h. Cellular β-sitosterol content was quantified by HPLC-MS and normalized to cell dry mass. Cell viability of Caco-2 (**b**) or MDCK.2 cells (**d**) was assessed after incubation with 0.75% oil or micellar oil for 24 h. Data were pooled from two independent experiments performed in duplicate. Bars represent mean ± SD. * *p* < 0.05.

In an initial analysis, β-sitosterol, campesterol, and stigmasterol were identified in the phytogenic oil at relative ratios of ~100:50:1 by HPLC-MS. Subsequently, differentiated Caco-2 cells were incubated with nonmicellar or micellar phytogenic oil, and the cellular content of β-sitosterol was quantitated. While β-sitosterol was not detected in untreated cells, incubation with phytogenic oils led to an increase in the β-sitosterol content. This effect was more pronounced after incubation with micellar oil (Figure 5a). Cellular stigmasterol levels were beyond the detection limit of HPLC-MS analysis, even after treatment with micellar or nonmicellar oil. Quantification of cellular campesterol levels was hindered due to partial overlap with an endogenous compound at *m/z* = 383, which remains to be identified. Neither the micellar oil nor nonmicellar oil interfered with cell viability (Figure 5b).

In MDCK.2 cells, a widely used model for epithelial cells, comparable effects were observed: β-sitosterol increased to a higher extent after incubation with the micellar phytogenic oil than after incubation with the nonmicellar oil, and neither of the oils interfered with cell viability (Figure 5c,d). Altogether, this indicates that micellization favors the cellular uptake of lipophilic compounds and that this effect is not restricted to fatty acids.

## 4. Discussion

We investigated the delivery of hydrophobic compounds from natural oils to target cells utilizing different cellular models. Micellar and nonmicellar algae oil was tested in cell models of enterocytes (Caco-2), epithelial cells (MDCK.2), and adipocytes (3T3-L1 and OP9). Micellization of the oil resulted in increased uptake of DHA and PUFAs compared to that of nonmicellar oil, an effect that was observed throughout the cell lines tested. This indicates that fatty acids from micellar oils are enriched intracellularly by mechanisms independent of lipases, lipid transporters, or lipid export mechanisms.

The amphiphilic nature of molecules necessary for micellization is of potential concern regarding adverse cytotoxic effects. Such effects were not apparent in the present study. Algae oils slightly decreased cell viability in Caco-2 cells, but only if applied for 24 h. The fact that this effect was comparable between the micellar and nonmicellar oils indicates that the reduction in cell viability is an adverse effect of lipid loading rather than being caused by the micellization process itself. Indeed, no adverse effect on cell viability was observed using micelles without algae oil alone (Supplementary Figure S1).

Micellar oil not only led to increases in DHA but also of α-linolenic acid and linoleic acid. However, the algae oil itself is low in these fatty acid species. DHA can be retro-converted to EPA via β-oxidation [29]. EPA itself is likewise rapidly catabolized, which ultimately leads to the formation of α-linolenic acid [30]. Therefore, the increase in α-linolenic acid in cell lines treated with micellar algae oil is likely caused by the partial catabolism of DHA. The increase in linoleic acid after incubation with micellar oil remains to be clarified because both algae oil preparations only contain 1–2% of this fatty acid.

Interestingly, the application of micellar oil significantly decreased relative arachidonic acid (ARA) levels in 3T3-L1 cells and tended to decrease relative ARA levels in Caco-2 cells. ARA is an ω-6 PUFA consisting of a 20-carbon chain fatty acid and an integral constituent of cell membranes, conferring it with fluidity and flexibility [31]. However, excess ARA is potentially harmful to human health because its endoperoxidation leads to the formation of bioactive eicosanoids including prostanoids and leukotrienes, which favor inflammatory processes [32]. In fact, high ARA intake has been shown to inhibit the anti-inflammatory and inflammation-resolving effects of ω-3 fatty acids [33]. Therefore, the observed increase in ω-3 fatty acids accompanied by a decrease in ARA may be beneficial, especially under pre-existing inflammatory conditions, such as cancer, cardiovascular disease, obesity, and diabetes [34].

Physiological absorption of long-chain fatty acids includes dispersion by bile acids, hydrolysis by pancreatic lipase, and absorption of monoglycerides and fatty acids. In healthy individuals, this process guarantees the uptake of 95–100% of fatty acids from the human diet. Under pathophysiological conditions of fat malabsorption, however, micellization of lipophilic compounds might become especially important for supplementation with essential fatty acids. Fat malabsorption occurs due to gastrointestinal diseases, such as Crohn’s disease [35], exocrine pancreatic insufficiency [36], cystic fibrosis [37], or after gastric bypass surgery [38]. Similarly, the use of Orlistat, an anti-obesity drug that limits fat absorption by inhibition of pancreatic lipase, leads to fat malabsorption [39]. Patients under this condition might benefit from the improved bioavailability of emulsified lipophilic nutrients, especially ω-3 fatty acids. Indeed, it was shown that emulsification increased the bioavailability of PUFAs, including DHA, in human subjects [40].

In addition to stimulating the uptake of PUFAs into target cells, we showed that phospholipid-based emulsification is also effective in increasing the uptake of phytosterols from phytogenic oils. In enterocytes, phytosterol absorption is regulated via uptake by Niemann-Pick C1-like protein 1 (NPC1L1), a process that is counteracted by resecretion of phytosterols via an ATP-dependent transporter heterodimer formed by ABCG5 and ABCG8 [41,42]. Both cell lines used in our study to monitor phytosterol uptake express NPC1L1; however, its surface activity is much stronger in MDCK.2 cells than in Caco-2 cells [43]. While Caco-2 express ABCG5 and ABCG8 [44], their presence has not yet been reported in MDCK.2 cells. In addition, a review of microarray data suggests that their expression is absent in MDCK.2 cells [45]. This implies that the cell lines tested in our study have strongly differing capabilities to handle phytosterols. Despite this, the delivery of phytosterols from micellar oil was equally effective in both Caco-2 and MDCK.2 cells, indicating that phospholipid-based emulsification does not rely on specific cellular phytosterol transport mechanisms. Of note, a previous study reported the successful emulsification of phytosterols using sucrose monolaurate, propylene glycol, and oleyl lactate [46].

## 5. Conclusions

Taken together, we investigated phospholipid-based emulsification as an approach to deliver fatty acids and phytosterols to a variety of cell types. We propose that this is a simple, effective, and safe approach that is also suitable for the delivery of other lipophilic compounds of interest to the desired target cells.

## Figures and Tables

**Table 1:** Total fatty acid composition of native and micellar algae oils.

Fatty acid	Formula	Configuration	Oil	Micellar Oil
			Relative Content	
lauric acid	C12:0	n.a.	0.08%	0.05%
myristic acid	C14:0	n.a.	0.35%	0.43%
myristoleic acid	C14:1	Z	0.30%	0.42%
C16:0	n.a.	11.12%	12.08%
palmitoleic acid	C16:1	Z	0.17%	0.25%
heptadecanoic acid	C17:0	n.a.	0.05%	0.05%
heptadecenoic acid	C17:1	Z	0.19%	0.17%
stearic acid	C18:0	n.a.	1.10%	1.23%
oleic acid	C18:1 n-9	Z	6.71%	5.85%
linoleic acid	C18:2 n-6	all Z	1.54%	1.94%
alpha-linolenic acid	C18:3 n-3	all Z	0.20%	0.23%
arachidic acid	C20:0	n.a.	0.07%	0.08%
eicosenoic acid	C20:1 n-9	Z	0.03%	0.03%
dihomo-gamma-linolenic acid	C20:3 n-6	all Z	0.11%	0.08%
arachidonic acid methyl ester	C20:4 n-6	all Z	0.09%	0.10%
eicosapentaenoic acid (EPA)	C20:5 n-3	all Z	0.42%	0.51%
behenic acid	C22:0	n.a.	0.11%	0.13%
decosapentaenoic acid (DPA)	C22:5 n-3	all Z	13.95%	11.97%
decosahexaenoic acid (DHA)	C22:6 n-3	all Z	63.41%	64.40%

n.a., not applicable.

## Supplementary Materials

The following are available online at https://www.mdpi.com/2072-6643/12/1/150/s1, Figure S1. Phospholipid-based micelles do not influence cell viability.
