# Supplementary material for: Increased Cellular Uptake of Polyunsaturated Fatty Acids and Phytosterols from Natural Micellar Oil
Source: Nutrients. 2020 Jan 5;12(1):150. doi: 10.3390/nu12010150 (PMC7019862; doi:10.3390/nu12010150)
Supplement: Supplementary file 1 [file nutrients-12-00150-s001.pdf]

Supplemental material to

Röhrl et al.

Increased cellular uptake of polyunsaturated fatty acids and phytosterols from natural micellar oil

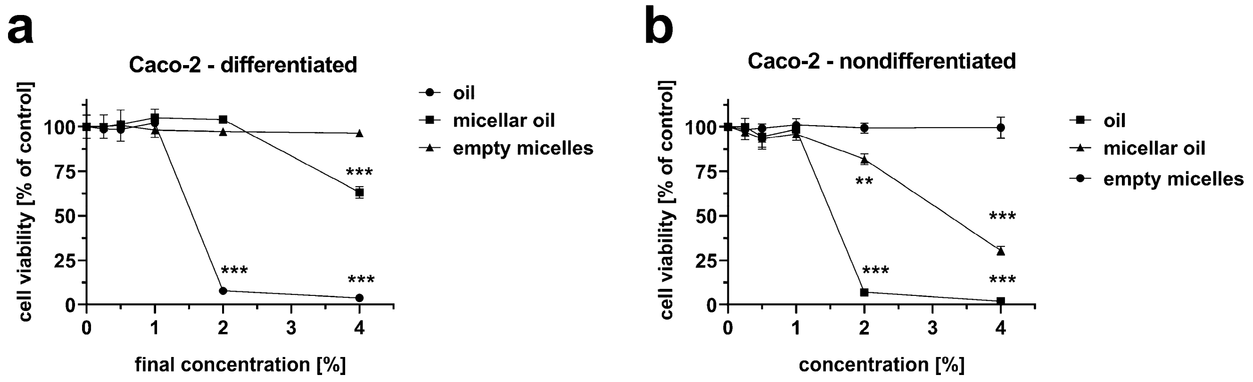

Figure S1. Phospholipid-based micelles do not influence cell viability. Differentiated (a) or nondifferentiated (b) Caco-2 cells were incubated with non-micellar oil ("oil"), micellar oil or empty micelles diluted in media containing 10% FBS at the indicated concentrations for 6 hrs. Micelles were produced by dispersion of phospholipids and glycerol in water by sonication. Cell viability was assessed by measuring conversion of resazurin to resurufin by metabolically active cells. Bars represent mean +/- SD.
